# Antioxidative Stress: Inhibiting Reactive Oxygen Species Production as a Cause of Radioresistance and Chemoresistance

**DOI:** 10.1155/2021/6620306

**Published:** 2021-02-08

**Authors:** Yanchi Chen, Yiling Li, Linyang Huang, Yu Du, Feihong Gan, Yanxi Li, Yang Yao

**Affiliations:** ^1^State Key Laboratory of Oral Diseases, West China Hospital of Stomatology, Sichuan University, Chengdu, China; ^2^West China School of Stomatology, Sichuan University, Chengdu, China

## Abstract

Radiotherapy and chemotherapy are the most effective nonsurgical treatments for cancer treatment. They usually induce regulated cell death by increasing the level of reactive oxygen species (ROS) in tumour cells. However, as intracellular ROS concentration increases, many antioxidant pathways are concurrently upregulated by cancer cells to inhibit ROS production, ultimately leading to drug resistance. Understanding the mechanism of antioxidant stress in tumour cells provides a new research direction for overcoming therapeutic resistance. In this review, we address (1) how radiotherapy and chemotherapy kill tumour cells by increasing the level of ROS, (2) the mechanism by which ROS activate antioxidant pathways and the subsequent cellular mitigation of ROS in radiotherapy and chemotherapy treatments, and (3) the potential research direction for targeted treatment to overcome therapeutic resistance.

## 1. Introduction

Reactive oxygen species (ROS) are derivatives of molecular oxygen formed by reduction–oxidation (redox) reactions or electronic excitation [1]. They are ubiquitous as by-products of chemical reactions in cell metabolism [2]. When the balance between ROS and antioxidants are disrupted, the body is under a state of oxidative stress [3]. This state may bring about inflammatory infiltration of neutrophils, increased secretion of proteases, and the production of large amount of oxidative intermediate products, all of which contribute to ageing and disease [4]. At excessive levels of intracellular ROS, cells take measures to clear ROS. These measures are called the antioxidant stress response [5]. ROS affect cell gene expression through various pathways. One classic pathway of cell resistance to ROS is the Keap1-Nrf2 system [6]. This system activates the transcription of a series of cytoprotective genes to increase the antioxidant level in the cell and reprogramme its metabolism to produce more glutathione and other substances. These effects can help the cell resist cell damage caused by ROS.

Radiotherapy and chemotherapy can kill tumour cells by several mechanisms, such as damaging DNA, increasing the ROS level, or damaging subcellular organelles [7, 8]. However, some tumour cells can survive these therapies and proliferate rapidly, limiting their therapeutic effect. This phenomenon is called therapeutic resistance. One way tumour cells develop this resistance is by increasing antioxidant levels and reprogramming metabolism to protect cells from the damage caused by ROS [4]. Some molecules receive signals of increased ROS in the cell, then enter the nucleus and react with some nucleic substances to regulate the transcription of some genes. The expression of these genes can increase the antioxidant level of the cell and reprogramme metabolism.

## 2. Killing Effect of Radiotherapy and Chemotherapy on Tumour Cells

### 2.1. Radiotherapy and Chemotherapy Increase Intracellular ROS Levels

In normal cells, ROS levels are kept low due to the antioxidant systems that maintain redox balance [9]. In cancer cells, the level of ROS increases to meet the need of malignant proliferation and progression but stays below the threshold to avoid cytotoxicity [10]. Radiotherapy uses radiation to irradiate tumour tissues and kill tumour cells. On the one hand, radiation acts directly on cells and instantly produces a large number of free radicals. On the other hand, it indirectly produces lasting and severe therapeutic effects through the redox reaction of water [11]. Due to the high content of water in cells, when water absorbs the energy of low-LET rays, a redox reaction occurs and a large number of free radicals and free electrons are produced. The free radicals and electrons generated initiate cascade reactions that produce OH, H_2_O_2_, and O_2_·^−^, significantly increasing the level of ROS [12]. Notably, oxidative changes can persist for several months after initial radiotherapy. This feature is related to the continuous generation of ROS and its heritability in the offspring of irradiated cells and obviously enhances the curative effect [13].

The mainstream treatment for cancer, chemotherapy, also often works by changing the redox state of cancer cells. Quite a few chemotherapeutics induce oxidative stress and ROS-mediated cell damage in cancer cells by increasing ROS above the threshold to yield an anticancer effect [14]. Most of these drugs produce ROS directly in cancer cells to increase the level of ROS. The first drug developed to achieve therapeutic effects by producing ROS was procarbazine. Procarbazine can be oxidised in aqueous solution and produce H_2_O_2_ and ·OH. When coordinating with ionising radiation, procarbazine forms unstable peroxides to damage DNA in vitro [15]. It was approved for the treatment of primary brain tumours and other diseases 60 years ago [16]. Nowadays, drugs like anthracycline [17] are widely used in cancer treatment to promote ROS production.

Another characteristic of the redox system in tumour cells is that it can increase the activity of ROS scavenging enzymes to adapt to internal oxidative stress [18]. Therefore, recently more attention has been focused on the inhibition of key molecules in the antioxidant system. For instance, sulfasalazine (an inhibitor of significant antioxidant glutathione) [19], chaetocin (an inhibitor of thioredoxin system) [20], and some other novel chemotherapeutic drugs are all targeted to inhibit the antioxidant system and increase the level of ROS.

### 2.2. ROS Are Responsible for Triggering Cell Death

Radiotherapy can directly cause DNA double-strand breaks through the immediate power of ionising radiation, thus blocking the cell cycle, preventing the proliferation of tumour cells, and eventually leading to cell death [21]. In addition, radiotherapy can cause indirect cellular effects, including bystander responses [22] and low-dose hypersensitivity [23], leading to a more extensive and lasting cell killing effect. These indirect reactions are related to the mechanism of cell death initiated by ROS [24]. Some of these indirect effects such as bystander responses have also been observed with chemotherapy [25]. Here, we primarily focus on how the increased ROS levels caused by radiotherapy and chemotherapy trigger cell death.

In normal conditions, ROS are maintained at a low dynamic balance under the effects of oxidation and antioxidation in cells [26]. Low levels of ROS are implicated in many intracellular chemical reactions and adjust the structure and function of proteins and lipids, whereas high levels of ROS damage DNA, proteins, and other cell components nonspecifically to hurt cells [9]. ROS levels can be divided into three types according to the effect on cells: (I) low level, normal physiological stage; (II) moderate level, carcinogenic stage; and (III) high levels, cell damage stage [5]. These stages present a gradual transition, and the dividing line of each process is not obvious. When ROS rise to moderate levels, they cause random mutations in cells due to DNA damage [27] and promote cell proliferation and metastasis [28], which exceeds the threshold of cell control and repair. These factors lead to the transformation of cells into cancer cells. ROS levels that continue to rise to high levels will lead to cell death. At this level, ROS trigger different types of regulated cell death, including apoptosis, autophagy, and ferroptosis.

#### 2.2.1. Apoptosis

Caspases, a family of proteases in cells, play an important role in apoptosis. They induce cell death by breaking down the key proteins in cells [29]. Caspases are activated by one of two pathways: death receptor-dependent pathway and mitochondrial-dependent pathway [30]. In the first pathway, the external apoptosis signal is triggered by death receptors on the cell surface and then activates caspase 8, producing a cascade reaction and finally leading to apoptosis [31]. ROS can induce apoptosis by regulating the expression of the death receptors and its ligand such as Fas-mediated apoptosis. Hydrogen peroxide (H_2_O_2_) promotes the expression of Fas by increasing its mRNA and protein levels [32]. In addition, it can upregulate the expression of death ligand Fas L and cause the activation of caspases 8 in Hela cells [33]. In the second pathway, mitochondria release caspase-activating proteins into the cytoplasm and cause apoptosis [34]. The permeability transition pore (PTP) on mitochondria plays a decisive role in this pathway [35]. Important regulators of the PTP opening are the Bcl-2 protein family, which can promote apoptosis [36]. ROS can oxidise and modify Bcl-2 proteins and then regulate apoptosis. B cell lymphoma-2 is an antiapoptotic member of the Bcl-2 protein family. Increased H_2_O_2_ induces the oxidative modification of B cell lymphoma-2 and downregulates its expression, promoting cell apoptosis [37].

#### 2.2.2. Autophagy

Autophagy maintains cell homeostasis by decomposing damaged organelles and proteins through lysosomes [38]. At a low ROS levels, autophagy can be induced by ROS and inhibited by antioxidants, and the three are in dynamic balance to maintain cell homeostasis [39, 40]. In oxidative stress, ROS damage DNA, lipids, and proteins and initiate autophagy [41]. At the same time, ROS molecules themselves also induce autophagy. The core of autophagy regulation is the Atg4 family of cysteine proteases. The activation of Atg4 is regulated by signal molecules including ROS, and H_2_O_2_ directly targets the oxidation of Atg4 to promote the formation of autophagosomes [42]. Once autophagy exceeds the limit of cell tolerance, it eventually causes autophagic death.

#### 2.2.3. Ferroptosis

Ferroptosis is a recently identified type of programmed cell death caused by lipid peroxidation [43]. Lipid peroxidation is a process in which ROS oxidise biofilms. ROS react with phospholipids, enzymes, and other macromolecules of biofilm to form lipid peroxidation products. It can directly change the fluidity and permeability of the cell membrane, destroy ion gradients, and affect the structure and function of cells [44]. In addition, the products of lipid peroxidation are highly bioactive, can disrupt the activity of DNA, proteins, and enzymes, and initiate cell death signalling pathways [45, 46]. Recent studies show that when radiotherapy induces increased intracellular ROS, the cells show the morphological characteristics of ferroptosis. Furthermore, the use of a ferroptosis inhibitor increases cell survival rates after radiation [47]. Therefore, high levels of intracellular ROS may directly promote ferroptosis by inducing lipid peroxidation.

**Figure 1:**
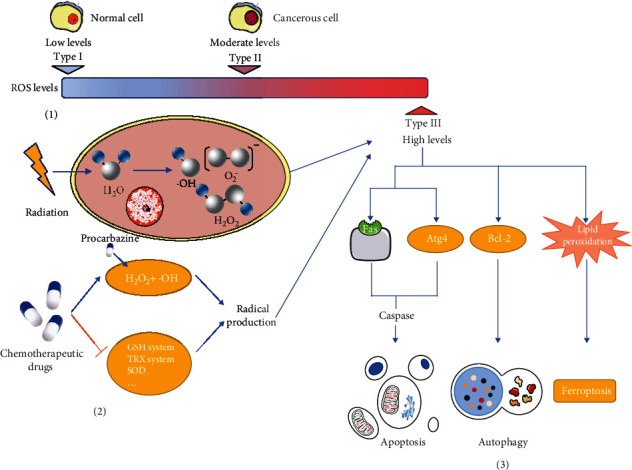
ROS are responsible for triggering cell death and the mechanisms of cancer treatment to trigger cell death. (1) ROS levels can be divided into three types according to their effects on cells. Type I is a low ROS level, wherein ROS only participate in normal cell physiological activities. Type II is a moderate ROS level, wherein ROS induce cell deformation within cancerous cells. Type III is a high ROS level, wherein ROS lead to cell death. (2) Radiotherapy and chemotherapy both increase the production of ROS. Radiotherapy causes the redox reaction of water and produces a large number of free radicals and free electrons. In chemotherapy, many drugs directly produce ROS in cancer cells to increase the level of ROS. (3) Radiotherapy and chemotherapy can cause indirect cellular effects by raising ROS levels to type III. High levels of ROS induce different types of regulated cell death. They regulate the expression of the death receptors such as Fas and Bcl-2 family proteins to induce apoptosis, target the oxidation of Atg4 to promote the formation of autophagosomes, which cause autophagic death, and directly promote ferroptosis by inducing lipid peroxidation.

The goal of radiotherapy and chemotherapy is to raise the level of ROS directly from type I/II to type III. And tumour resists the therapy by maintaining moderate ROS levels at type II. This is also the basic reason why radiotherapy and chemotherapy can achieve the purpose of tumour treatment and why tumours develop therapeutic resistance (Figure 1).

## 3. Antioxidative Stress-Related Pathways That Lead to Therapeutic Resistance

As prooxidant cancer therapy increases the effective ROS concentration in cancer cells, irreversible oxidative stress is generated that damages DNA, lipids, and proteins, causing the suppression of cancer cells [48, 49]. However, many cell signalling pathways that adapt to prooxidant therapy-induced oxidative stress are activated by this increased ROS. These pathways have various functions, such as increasing antioxidant levels and reprogramming metabolism [50], which can inhibit ROS production to adapt to oxidative stress and produce therapeutic resistance.

### 3.1. Keap1-Nrf2 Signalling Pathway

#### 3.1.1. Activation of the Keap1-Nrf2 Signalling Pathway under Oxidative Stress

**Figure 2:**
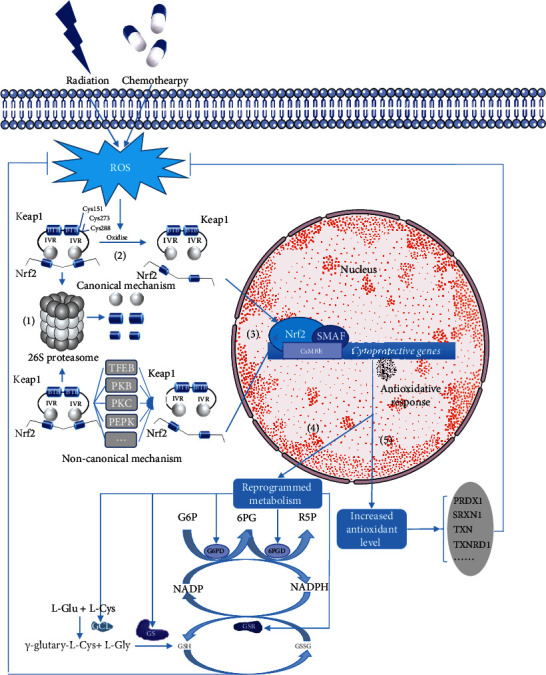
The role of the Keap1-Nrf2 signalling pathway in antioxidative stress-related radio- and chemoresistance. (1) Degradation of Nrf2. Under normal conditions, Keap1 combines with Nrf2 to promote the degradation of Nrf2 by the 26S proteasome. (2) Activation of Nrf2. Radiation and chemotherapies increase the effective ROS concentration in cancer cells via the redox reaction of water and direct production of ROS, respectively. Later, increased ROS oxidise three cysteines within Keap1 to activate Nrf2 by dissociating Nrf2 from Keap1 and slowing down the speed of Nfr2 degradation. (3) Translocation of Nrf2. Activated Nrf2 translocates into the nucleus, forming the Nrf2-sMAF heterodimer with one of the small MAF (sMAF) proteins. The Nrf2-sMAF heterodimer binds to the CNC-sMAF binding element (CsMBE), resulting in increased antioxidant levels and reprogrammed metabolism. (4) Increased antioxidant enzymes. Various antioxidant genes including peroxiredoxin 1 (*PRDX1*), sulfiredoxin 1 (*SRXN1*), thioredoxin (*TXN*), and thioredoxin reductase 1 (*TXNRD1*) can be activated by Nrf2. (5) Reprogrammed metabolism. G6PD and 6PGD are the two major enzymes in the pentose phosphate pathway (PPP), which generates NADPH. Glutamate cysteine ligase (GCL) and glutathione synthase (GS) are the two rate-limiting enzymes in glutathione (GSH) de novo synthesis. Nrf2 promotes the translation and expression of GCL, GS, GSR, G6PD, and 6PGD. Increased antioxidant enzymes and reprogrammed metabolism can protect cancer cells from ROS-triggered cell death, leading to radio- and chemoresistance.

The Keap1-Nrf2 signalling pathway performs a significant role in cell protection and adaptation against oxidative stress. This pathway comprises two main parts: Kelch-like ECH-associated protein 1 (Keap1) and nuclear factor erythroid 2-related factor 2 (Nrf2) [51]. Under normal physiological conditions, Keap1 interacts specifically with Cullin 3 (Cul3) and forms an E3 ligase complex to stimulate the ubiquitination of Nrf2, ultimately leading to the targeted degradation of Nrf2 by the 26S proteasome [52, 53] (Figure 2). In this case, the Nrf2 protein is inactivated and has a short half-life. Thus, the activation of the Keap-Nrf2 signalling pathway is prevented.

However, in case of radiotherapy or chemotherapy-meditated oxidative stress, Nrf2 can be activated by detaching from Keap1. With radiation and drugs increasing the level of ROS in cells, the cysteines which residues in Keap1 and function as redox sensors are oxidised causing the dissociation of Keap1 between Nrf2 and slowing down the speed of Nfr2 degradation. Three functionally important cysteines that regulate the activation of Keap1-Nrf2 have been found: Cys151, Cys273, and Cys288 [54]. Among them, Cys273 and Cys288, both of which reside in the region of the IVR domain, are critical for Keap1 to inhibit Nrf2 under normal conditions, whereas a subset of Nrf2 activators target Cys151 which locate in the BTB domain [55, 56]. Recent findings have shown that the modification of Cys151 residue in Keap1 is crucial in activating Nrf2 by artemisitene and curcumin [56, 57] (Figure 2). Apart from the canonical mechanism to activate Nrf2 by oxidising Keap1 cysteine, other noncanonical Nrf2 regulatory pathways have been found under stressful conditions. These include proteins containing an ETGE amino acid motif such as sequestosome 1/p62, dipeptidyl peptidase 3 [58]; kinases including protein kinase B (PKB/AKT, see Section 3.2.3), protein kinase C extracellular-regulated protein kinases [59], protein kinase-like endoplasmic reticulum kinase [51]; transcriptional factor EB [60]; and acetyltransferase p300 [61] (Figure 2). As Nrf2 and Keap1 detach, Nrf2 becomes dissociated and transferred to the nucleus to induce the adaptation to oxidative stress.

#### 3.1.2. Activated Nrf2 Induces ROS Mitigation

Activated NRF2 translocates into the nucleus and interacts with one of the small MAF (sMAF) proteins, forming the Nrf2-sMAF heterodimer [62, 63]. The heterodimer plays an important role in inducing the expression of cytoprotective genes, leading to radio- and chemoresistance in tumour cells. It binds to DNA sequences referred to as the antioxidant response element [64] or electrophile response element [65], now collectively defined as the CNC-sMAF binding element (CsMBE) [66]. Most of these sequences are cytoprotective genes. In addition, the way that these heterodimers bind to CsMBE is stress dependent [63] (Figure 2). The binding of the Nrf2-sMAF heterodimer and CsMBE primarily results in increased antioxidant levels and reprogrammed metabolism.

*(1) Nrf2-sMAF and Increased Antioxidant Levels*. The antioxidant function of Nrf2-sMAF is the most canonical way that Nrf2 promotes the adaptation of cancer cells to oxidative stress as it activates the transcription and translation of a number of antioxidant enzymes or proteins in a stress-dependent manner [67]. For the last two decades, using genome-wide chromatin immunoprecipitation analysis, scientists have targeted various Nrf2-dependent antioxidant genes including peroxiredoxin 1 (*PRDX1*), sulfiredoxin 1, thioredoxin, and thioredoxin reductase 1 [68–70]. Most of the related enzymes can be directly activated by Nrf2-sMAF; here, we primarily focus on PRDX1 as an example of the function of antioxidant enzymes. Peroxiredoxins (PRDXs) are a highly conserved family of peroxidases that reduce ROS [71]. PRDX1 is one of the 2-Cys PRDXs subfamily members that has been reported to have potential radio- and chemoprotective effects [72–74]. The established mechanism is that PRDX1 detoxifies H_2_O_2_ by reducing it with the thioredoxin (TRX) system and supplying reducing equivalents [75]. However, PRDX1 may also play its antioxidant role by affecting ROS-dependent signalling pathways [72]. In one study, the downregulation of PRDX1 in lung cancer cells was found to reverse radioresistance and enhance radiosensitivity [76].

*(2) Nrf2-sMAF and Reprogrammed Metabolism*. Recent analyses show that Nrf2 contributes to stress adaptation by regulating intermediary metabolic pathways [77]. In addition, reprogrammed metabolism has been found to be closely related to the development of radio- and chemoresistance [78]. Some metabolic enzyme modulating sequences were identified as Nrf2-sMAF target genes including those involved in glutamine and glucose metabolism [77, 79, 80].

Glutathione (GSH) functions as antioxidant defence and plays an important role in maintaining the redox homeostasis in cells. Nrf2 is thought to be a critical transcriptional controller of GSH metabolism by regulating a series of enzymes of GSH metabolism including the GSH de novo synthesis enzymes glutamate cysteine ligase (GCL) and glutathione synthase (GS), as well as the GSH regeneration enzyme glutathione reductase (GSR) [81, 82]. Specifically, Nrf2-sMAF controls the expression of catalytic and regulatory subunits of the rate-limiting enzyme complex, which are the major determinants of glutathione synthesis [83]. Besides the enzymes that control the glutamine metabolism directly, a cystine/glutamate exchange transporter called system X also plays a critical role in intracellular GSH biosynthesis [84]. The expression of the light chain of system X is promoted by Nrf2 under the stimulation of oxidative stress, which is closely related to radio- and chemoresistance in tumour cells [85, 86]. In Nrf2 knockdown cells, glutathione metabolism is affected [80], showing the pivotal role of Nrf2 in glutathione metabolism.

The glucose metabolism phenomenon of cancer cells known as the Warburg effect indicates the paradoxical fact that most tumour cells rely on aerobic glycolysis even in an oxygen environment [87, 88]. Due to the Warburg effect, cancer cells have more glucose uptake than normal cells. When large amounts of glucose enrich cells, the pentose phosphate pathway (PPP), which generates NADPH, dominates [89]. NADPH is critical in cellular antioxidation systems and protects the cell from oxidative stress [89, 90]. Nrf2-sMAF mostly controls the production of NADPH via the PPP. The activation of Nrf2 signalling in cancer cells promotes the expression of PPP genes by weakening miR-1 and miR-206 expression, leading to the reprogramming of glucose metabolism [91].

The expression of some major enzymes of PPP is Nrf2 dependent. The increased expression and activity of glucose-6-phosphate dehydrogenase (G6PD), which is the first and rate-limiting enzyme in the PPP, is promoted by Nrf2 [92]. In addition, the overexpression of 6-phosphogluconate dehydrogenase (6PGD), the third oxidative decarboxylase of the PPP in cancer cells, is mediated by Nrf2 [78].

Another fact that should be seriously considered is the relationship between GSH, NADPH, and the antioxidant reaction. As the intracellular ROS level increases (e.g., H_2_O_2_), GSH can eliminate H_2_O_2_ and turn it into water under the catalysis of glutamate peroxidase. At the same time, GSH is converted to its oxidised form, GSSG. GSH is then reduced from GSSG by NADPH under the catalysis of GSR to detoxify ROS entirely. As mentioned above, the generation of GSH, the GSH regeneration enzyme, and the production of NADPH are all controlled by Nrf2, demonstrating the important role of activated Nrf2 in the cellular response to oxidative stress (Figure 2).

### 3.2. PI3K-AKT Signalling Pathway

#### 3.2.1. Activation of the PI3K-AKT Signalling Pathway under Oxidative Stress

The intricacies of the PI3K-AKT signalling pathway have already been reported by previous reviews in detail [93, 94]. Here, we primarily focus on the ROS-dependent activation of PI3K-AKT. When receptors are activated by their ligands such as G-protein-coupled receptors, it stimulates the recruitment of class 1 phosphoinositide-3-kinases (PI3Ks), which ultimately activate PI3K. Later, the activated PI3K phosphorylates phosphatidylinositol 4,5-bisphosphate (PIP2) to phosphatidylinositol 3,4,5-trisphosphate (PIP3) [95]. As PIP3 accumulates, it acts as a membranal signalling molecule and subsequently recruits and activates protein kinase B (PKB/AKT) [51, 96]. The serine–threonine kinase AKT is one of the most important downstream effectors of PI3K signalling, which controls a large number of pathways. When AKT binds to PIP3, it is activated with phosphoinositide-dependent protein kinase 1 and rapamycin complex 2, phosphorylating the T308 and S473, respectively [97]. Moreover, the primary functional antagonist of PI3K, phosphatase and tensin homolog (PTEN), inhibits the activation of AKT by dephosphorylating PIP3 to PIP2 [98].

**Figure 3:**
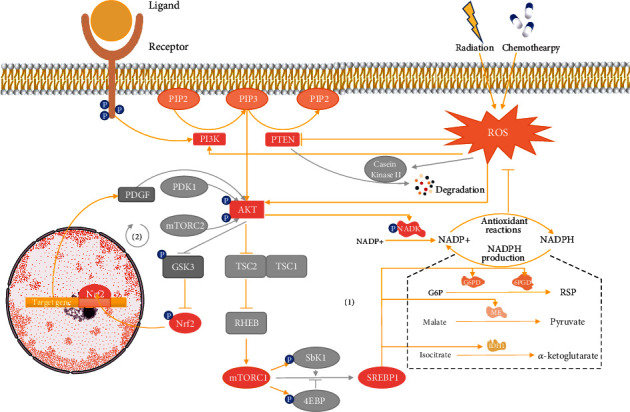
(1) The role of the PI3K-AKT signalling pathway in antioxidative stress related to radio- and chemoresistance. Phosphoinositide-3-kinases (PI3Ks) phosphorylate phosphatidylinositol 4,5-bisphosphate (PIP2) to phosphatidylinositol 3,4,5-trisphosphate (PIP3), which subsequently recruits and activates protein kinase B (PKB/AKT). However, these processes can be inhibited by phosphatase and tensin homolog (PTEN), which dephosphorylates PIP3 to PIP2. As radiation and chemotherapy continuously generate ROS via the redox reaction of water and direct production of ROS, respectively, increased intracellular ROS activate the PI3K-AKT pathway by directly promoting PI3K and AKT and inhibiting PTEN. PI3K-AKT signalling serves as defence against ROS by promoting NADPH production. Activated AKT regulates NADPH metabolism in direct and indirect ways via NAD^+^ kinase (NADK) and rapamycin complex 1 (mTORC1), respectively. NADK is the unique cytosolic enzyme that catalyses the phosphorylation of NAD^+^ to NADP^+^, enlarging the size of the NADP^+^ and NADPH pool. Downstream of mTORC1, sterol regulatory element-binding protein (SREBP) stimulates the expression of G6PD, 6PGD, IDH1, and ME, reducing NADP^+^ to NADPH. (2) The loop formed between AKT and Nrf2. Glycogen synthase kinase 3 (GSK3) and platelet-derived growth factor (PDGF) act as a linker to connect AKT with Nrf2. PI3K-AKT signalling inhibits the Keap1-independent degradation of Nrf2 by phosphorylating GSK3. Activated Nrf2 translocates to the nucleus and upregulates the transcription of PDGF, binding with its cognate receptors to stimulate AKT.

As radiotherapy and chemotherapy change the redox state of cancer cells, the increased ROS has the ability to activate PI3K or AKT directly to amplify the downstream of PI3K-AKT signalling. Meanwhile, PTEN is inhibited, promoting the activation of PI3K-AKT [96]. Increased ROS not only oxidise the cysteine residue located in the active centre to modulate PTEN directly but also promote the phosphorylation of serine/threonine within the C-terminus of the protein by casein kinase II [98, 99]. The phosphorylation of PTEN prevents its recruitment to the membrane and promotes its ubiquitination, ultimately leading to the proteolytic degradation pathway [96, 98] (Figure 3).

#### 3.2.2. Activated AKT Induces ROS Mitigation

With further exploration of downstream effectors that mitigate ROS levels, the PI3K-AKT signalling pathway was recently found to induce the adaptation to oxidative stress separate from the Keap1-Nrf2 pathway [97]. The principal mechanism of PI3K-AKT to reduce ROS levels is to reprogramme metabolism, which promotes the production of NADPH. Activated AKT not only phosphorylates metabolic enzymes directly but also regulates several downstream effectors, among which mammalian target of rapamycin complex 1 (mTORC1), and Nrf2 (see Section 3.2.3) seems to play an important role [97].

The direct method by which AKT promotes NADPH production is closely related to NAD^+^ kinase (NADK), a unique cytosolic enzyme that catalyses the phosphorylation of NAD^+^ to NADP^+^ using the magnesium ion as a cofactor and ATP as the phosphate donor [100, 101]. AKT directly stimulates the activation of NADK by phosphorylating three serine residues (Ser44, Ser46, and Ser48) within the N-terminal region [102]. Then, activated NADK promotes the production of NADP^+^, which is subsequently reduced to NADPH. The synthesis of NADP^+^ from NAD^+^ via NADK enlarges the size of the NADP^+^ and NADPH pool, which may resist the loss of oxidised NADPH and result in the adaptation to increased ROS level.

The mTORC1 has been reported to play an important role in producing NADPH and has received much attention as one of the downstream substrates of the PI3K-AKT signalling pathway. The tuberous sclerosis (TSC) 1 and TSC2 functional complex is the intermediate regulator of the PI3K-AKT-mTORC1 pathway. Under normal conditions, the complex inhibits mTOR, which meditates the inhibition of p70 ribosomal protein S6 kinase 1 (p70S6K, also S6K1) and the activation of eukaryotic initiation factor 4E binding protein 1 (4EBP1) [103]. As mentioned above, when intracellular ROS levels are increased by radiotherapy and chemotherapy, AKT becomes activated. Activated AKT directly phosphorylates TSC2, disrupting and inactivating the TSC1-TSC2 complex. The destabilisation of TC2 promotes Ras homolog enriched in brain activity, ultimately activating mTORC1 [104]. Thus, the two canonical key downstream proteins S6K1 and 4EBP1 are all phosphorylated by mTORC1, leading to activation and inactivation, respectively [105]. S6K1, the major substrate protein molecule of mTORC1, promotes the activation of sterol regulatory element-binding protein (SREBP) [106].

In the cytoplasm, the most important pathway that reduces NADP^+^ to NADPH is the oxidative PPP with G6PD and 6PGD catalysing the key steps. In addition, isocitrate dehydrogenase 1 (IDH1) and malic enzyme (ME) can regenerate NADPH from NADP^+^. IDH1 and ME catalyse the oxidative decarboxylation of isocitrate to *α*-ketoglutarate and of malate to pyruvate, respectively, ultimately reducing NADP^+^ to NADPH. SREBP can promote the production of NADPH by stimulating the expression of these four enzymes. The mRNAs for G6PD, 6PGD, and ME were found to be elevated in SREBP-overexpressed transgenic mice, indicating that these three enzymes are potentially activated by the ROS-meditated upregulation of SREBP [107]. Upregulated SREBPs bind with the IDH1-SRE sequence element GTGGGCTGAG within the promoter region to activate IDH1 [108]. Using 25-hydroxycholesterol or stains to inhibit or activate SREBP, respectively, Ricoult et al. demonstrated SREBP-mediated regulation on IDH1 expression [109]. The complicated mechanism of PI3K-AKT-mediated production of NADPH protects cancer cells against ROS, potentially revealing a new aspect of radio- and chemoresistance (Figure 3).

#### 3.2.3. Crosstalk between PI3K-AKT and Keap1-Nrf2 Signalling Pathways

Several studies have covered the interaction between PI3K-AKT and Keap-Nrf2 signalling pathways. For example, using the PI3K inhibitor LY294002 to repress the PI3K-AKT pathway inhibits the nuclear translocation of Nrf2 [110]. Nrf2 regulates metabolic reprogramming, and its function can be expanded by the continuous activation of the PI3K-AKT pathway [80]. He et al. found that in human hepatomegaly, all the factors including oxidative stress and liver cancer that stimulate Nrf2 result in the activation of AKT [111]. Interestingly, it seems that the PI3K-AKT and Keap-Nrf2 pathways form a loop so that both can act as the downstream effector of the other. Glycogen synthase kinase 3 (GSK3) is the intermediate factor of the PI3K-AKT-Nrf2 pathway and mediates the Keap1-independent degradation of Nrf2 [112]. GSK3 phosphorylates NRF2 to stimulate the ubiquitination of Nrf2, which is subsequently marked for proteasomal degradation [113]. Given that the phosphorylation and inhibition of GSK3 are mediated by AKT activation [114], the activation of the PI3K-AKT pathway could promote the stabilisation of Nrf2 by inhibiting GSK3. By contrast, Nrf2 can stimulate the activation of AKT. When Nrf2 translocates to the nucleus, it recruits specificity protein 1 to the promoter of platelet-derived growth factor (PDGF) to upregulate its transcription [115]. In addition, the activation of AKT is closely related to the binding of PDGF and its cognate receptors [111]. To conclude, PI3K-AKT controls Nrf2 indirectly via the downstream kinase GSK3, whereas Nrf2 promotes the activation of AKT at transcriptional and translation levels through PDGF (Figure 3).

### 3.3. Prooxidant Therapy and Antioxidant Pathways

**Figure 4:**
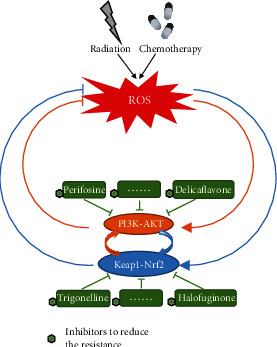
Effective treatment methods to overcome therapeutic resistance. PI3K-AKT and Keap1-Nrf2 signalling pathways are activated after radio- or chemotherapy for ROS production. The downstream antioxidant elements of these two pathways facilitate cancers with radio- and chemoresistance by resisting the cytotoxicity of high ROS. Halofuginone, trigonelline, delicaflavone, and perifosine are potential inhibitors of the PI3K-AKT or Keap1-Nrf2 pathway to reduce resistance and meet the need of radiosensition and chemosensition.

It is generally acknowledged that ROS are one of the primary mediators of ionising radio- and chemotoxicity, which leads to the death of cancer cells. Radiation and some chemotherapy drugs trigger tumour cell death by upregulating ROS to the threshold needed to treat tumours. However, while prooxidant therapy increases intracellular ROS levels to treat the patient, many antioxidant pathways have also been activated to interfere with oxidative stress due to increased level of ROS, giving rise to radioresistance and chemoresistance. NADPH serves as the most significant reducing agent to antioxidant defence systems, which protect tumour cells from the cytotoxicity of ROS. ME- and IDH-dependent NADPH production and the oxidative PPP are the three primary pathways that enlarge the cytosolic NADPH pool. As mentioned above, the continuous generation of ROS from radio- and chemotherapy activates Keap1-Nrf2 and PI3K-AKT pathways, which regulate several antioxidative downstream effects. Both of these signalling pathways upregulate the expression of some major enzymes of the PPP including 6PGD and G6PD. Furthermore, the PI3K-AKT pathway stimulates the expression of ME and IDH, contributing to the regeneration of NADPH from NADP^+^ via the middle effector SREBP. Apart from promoting the production of NADPH, the activated antioxidant pathways also increase antioxidant levels to mitigate ROS. Based on this mechanism, it is easy to conceive that as the Keap1-Nrf2 or PI3K-AKT signalling pathway is activated by increased ROS, cancer cells are facilitated with the ability to resist the prooxidant therapy-meditated generation of ROS, resulting in radio- and chemoresistance. In fact, several inhibitors of these two signalling pathways have been found, including halofuginone [116], trigonelline [117], delicaflavone [118], and perifosine [119], which are potential therapeutic strategies to weaken radioresistance and chemoresistance (Figure 4). Trigonelline, an effective inhibitor of Nrf2, was demonstrated to overcome oxaliplatin resistance in colon cancer cells [117]. Delicaflavone may potentially break down the therapy resistance in colorectal cancer by the significant inhibition of the phosphorylation levels of the PI3K-AKT signalling pathway and the subsequent generation of ROS [118] (see Section 4 for more details). Thus, the Keap1-Nrf2 and PI3K-AKT pathways should be considered for radiosensitivity and chemosensitivity.

## 4. Role of ROS in Radiosensition and Chemosensition

While overproduced ROS adapt to the increased metabolism of tumour cells, high ROS levels caused by radiotherapy and chemotherapy also result in cytotoxicity, indicating a close connection between ROS levels and the sensitivity of cells to treatment [120–122]. To improve the sensitisation of tumours, much attention has been paid to targeted therapies that interfere with the changes in ROS levels, including generation, degradation, and regulation pathways [123]. Generally, current research has primarily focused on three aspects: (1) regulating the generation and elimination of ROS [124], (2) adjusting metabolism [125], and (3) ameliorating hypoxic environment [126]. Each aspect has its own unique treatment mechanism which is aimed at its target pathways. Therefore, to explore effective treatment methods to overcome therapeutic resistance, it is necessary to gain a full understanding of the key principles and their influences on redox homeostasis. Moreover, the treatment needs to be applied to the whole body to determine its effectiveness. This effectiveness should also consider the advantages and disadvantages between the damage of normal tissue and the killing of tumour tissue.

### 4.1. Regulating the Generation and Elimination of ROS

Active metabolism in tumour cells leads to high production of ROS. To resist the cytotoxicity of high ROS, antioxidant systems are activated to maintain ROS at a relatively secure level [127]. To enhance their sensitivity, tumour cells increased ROS levels by promoting the production of ROS or inhibiting the antioxidant system. As an important source of ROS, mitochondrial dysfunction is the primary reason for increased ROS production in cancer cells and provides a chief focus for targeted therapy [128]. Currently, targeted therapeutic drugs (e.g., elesclomol [129] and rotenone [130]) for ROS production mostly focus on the mitochondrial electron transport chain as a specific inhibitor to the complex. The increase of ROS production leads to apoptosis and ultimately is manifested as tumour sensitisation. In addition, some special ROS molecules produced through relatively independent pathways, such as nitric oxide (·NO), offer a completely different treatment idea. Although ·NO is inert in most cases, its reaction rate with O_2_·^−^ in cells is even faster than that of O_2_·^−^ disproportionation catalysed by SOD [131]. In this way, directly providing donors or regulating synthetases may be novel methods to increase the level of ROS [132, 133].

For the antioxidant system, due to the numerous pathways involved, more options are provided for the selection of inhibitors. The first choice at present is undoubtedly the direct inhibition of several important pathways, such as the pathways mentioned above (Figure 4). For the Keap1-Nrf2 pathway, halofuginone was reported to be able to deplete all Nrf2 in cells by inhibiting all protein synthesis, thus reducing the drug resistance of tumour cells in vivo and in vitro [116]. In addition, trigonelline is a potent inhibitor of Nrf2 and causes a higher repression on the expression of the downstream antioxidant response element [117]. Recent studies have indicated that trigonelline is a potential inhibitor to reduce resistance in the treatment of colon cancer and hepatocarcinoma [117, 134]. For the PI3K-AKT pathway, delicaflavone significant inhibited resistance and induced apoptosis effectively [118]. However, because the antioxidant system is complex, regulating its related factors can also indirectly inhibit the pathway. Cyclooxygenase-2 overexpression has been confirmed to mediate the activation of the PI3K-AKT pathway and is associated with drug resistance in non-small-cell carcinoma [135]. Moreover, cyclooxygenase-2 participates in the regulation of the NF-*κ*B pathway, suggesting another method for regulating ROS elimination [136].

### 4.2. Adjusting Metabolism

Tumour cells reprogramme metabolism to meet the need of malignant proliferation and metastasis [125]. The altered metabolism provides more sources for the increase in ROS, improving the resistance of the tumour. Pavlova et al. divided the known metabolic characteristic changes in tumours into six groups according to their effects on cell genes, differentiation, and the microenvironment [137]. While most cancers often display a few of these effects, the grouping provides a clearer direction for the research of treatments for a certain type of tumour. Among these groups, two are considered to be closely related to the change in ROS levels. One is the increase in glucose and glutamine catabolism. As two major nutrients for cell survival and biosynthesis, glucose and glutamine are consumed by tumour cells at a significantly increased rate [138, 139]. To adapt to these changes, tumour cells strictly control their own state by reprogramming the metabolic process [140]. Different metabolic modes also cause the increase in ROS. This provides a direction for targeted therapy. As previously mentioned, Nrf2 affects cell metabolism. As a targeted inhibitor of Nrf2, 2′,4′-dihydroxy-6′-methoxy-3′,5′-dimethylchalcone can significantly decrease GSH content and GST activity [141]. In addition, the inhibitor K-563 produced by Streptomyces sp. is able to reduce the production of GSH by inhibiting the Keap1-Nrf2 pathway [142]. Both inhibitors raise the sensitivity of tumour cells by adjusting glucose and glutamine catabolism.

The other related group is the change in the use of intermediate products in the tricarboxylic acid cycle. Tumour cells not only have increased demand for nutrients but also change the using ways of nutrients [88]. The variation in redox reactions of the tricarboxylic acid cycle supplies more opportunities for ROS induction. The key mechanism of NADPH oxidase 4 promoting cell growth is considered to be the PI3K/AKT pathway in the antioxidant system, indicating the connection between ROS levels and reprogramming metabolism [143]. Targeted therapy to inhibit the pathways related to these aspects is expected to be beneficial to the regulation of cellular ROS levels.

### 4.3. Ameliorating Hypoxic Environment

As a marker of the tumour microenvironment, hypoxia greatly reduces the sensitivity of tumour cells to effective treatment. Various changes in cells caused by hypoxia promote the development of the tumour and the generation of therapeutic resistance [144]. In the process of the cell response, the HIF family plays a significant role as transcription factors [145]. Therefore, it is easy to think that the inhibition of HIF by targeted therapy would achieve a good prognosis. FTY720 (Fingolimod) [146], L-carnosine dipeptide [147], and LW6 [148] are inhibitors of HIF1 that are currently being studied. They primarily repress therapeutic resistance by inhibiting the accumulation of HIF factors, thus reducing the expression of target genes. However, although the inhibition therapy is the most direct and effective treatment in theory, the current treatment results are not ideal due adverse effects and low bioavailability [149]. At present, effective targeted therapy drugs are still under exploration.

Because tumour therapies are less effective due to hypoxia, another approach that has recently attracted attention is the combination of a nanosensitiser and traditional treatment, which is aimed at relieving the anoxic environment while delivering drugs. For instance, one study reported that a special nanoparticle consisting of doxorubicin (DOX) and a MnO shell can be delivered to tumour tissues and released by a near-infrared laser, then resolve HO to ameliorate the hypoxia environment [150]. Moreover, when the nanoparticles contain different materials, they play different auxiliary roles in diverse tumour treatment. For example, novel shell-stacked nanoparticle can wrap and deliver targeted medicine to tumour cells and neovascularization [151]. A prodrug system made up of hybrid nanoenzymes may improve hypoxic conditions in tumour cells and provide better basis for chemophotodynamic treatment [152]. Nanotechnology provides another possible method to stimulate the sensitivity of tumour cells and overcome therapeutic resistance.

### 4.4. Precise Regulation of ROS Levels under Systemic Condition

Although the inhibition of antioxidants can improve the therapeutic effects of adjuvant radiotherapy and chemotherapy, the role of the antioxidant system in normal tissue cannot be ignored. In most cases, the antioxidant system protects normal tissue by scavenging ROS [127]. Studies have shown that antioxidants play a significant role in protecting and preventing carcinogenesis [153]. As an antioxidant, isoflavone inhibits the activation of NF-*κ*B in oxidative stress [154]. Due to its antioxidant ability, isoflavone can inhibit the production of H_2_O_2_ caused by tumour promoters *in vitro* and *in vivo*, indicating its potential ability to prevent carcinogenesis [155]. In addition, the antioxidant eugenol can effectively inhibit lipid peroxidation and reduce iron and copper ions, suggesting its strong antioxidant activity and free radical scavenging ability [156]. Eugenol has been shown to play a role in preventing skin cancer and reducing the incidence of gastric cancer, the actions of which are closely related to its antioxidant activity [157, 158]. However, some studies indicate that excessive antioxidation also has harmful effects on normal cells. In clinical trials, people taking antioxidant supplements have shown a higher risk of developing skin cancer [159, 160]. The antioxidant epigallocatechin gallate can induce DNA double-strand breaks in human lung and skin normal cells, resulting in DNA damage and cell death [161]. Moreover, high doses of synthetic antioxidants, which eliminate the interference of cytotoxicity, have been demonstrated to cause DNA damage in cultivated mesenchymal stem cells, finally inducing premature senescence [162].Therefore, only antioxidants maintained within a certain concentration range can be beneficial to normal issue.

However, this does not mean that the idea of inhibiting antioxidants to enhance radiotherapy and chemotherapy is wrong. On the contrary, radiation protective agents such as the antioxidant 3,3′-diindolylmethane make use of the characteristic to reduce the damage to normal tissue caused by radiotherapy. 3,3′-Diindolylmethane can inhibit the accumulation of ROS and has strong free radical scavenging activity [163]. In addition, it prevents tumorigenesis by protecting DNA from damage in colon cancer [164]. Furthermore, it has been proposed as a radioprotective drug because it protects normal tissue from radiation damage [165]. Other representative drugs such as the cysteamine series also have strong protective effects [166]. Amifostine, which is widely used in clinical practice, significantly reduces the effect of radiotherapy on normal tissue by scavenging free radicals [167]. In fact, the ideal therapeutic method is to enhance the effect of antioxidants in normal tissue and suppress it in tumour tissue.

## 5. Conclusion

ROS are the redox products of cellular metabolism. Under normal conditions, the production and clearance of ROS are in balance to keep them at a stable low level. In tumour cells, the appropriate increase of ROS plays an important role in the malignant progression of cancer. However, high levels of ROS can also induce regulated cell death such as apoptosis, autophagy, and ferroptosis by affecting cell signalling pathways and promoting lipid peroxidation. As primary antitumour treatment methods, radiotherapy and chemotherapy primarily increase the level of ROS to achieve their therapeutic effect. However, in the process of treatment, tumour cells correspondingly enhance antioxidant stress to prevent ROS levels from being too high. Here, we review some important mechanisms of antioxidant stress in tumour resistance to radiotherapy and chemotherapy. Previous studies have shown that some important pathways, such as Keap1-Nrf2 and PI3K-AKT, are significantly upregulated to reduce ROS levels by increasing antioxidant levels, changing metabolism, or other mechanisms. These findings suggest that the inhibition of ROS production is an important reason for the therapeutic resistance of tumour cells. In this context, treatment targeting the antioxidative stress system is an important research direction to overcome radioresistance and chemoresistance. Prior research indicates that ROS levels can be increased by directly promoting ROS production, inhibiting antioxidant system, regulating metabolism, and ameliorating hypoxia environment. At present, the relevant research is still in the state of exploration but shows great potential for future study of the tumour cell antioxidant stress system.
